# Drug efficacy on zoonotic nematodes of the Anisakidae family: new metabolic data

**DOI:** 10.1017/S0031182022000543

**Published:** 2022-07

**Authors:** Iwona Polak, Robert Stryiński, Magdalena Podolska, Joanna Pawlak, Mikołaj Wiktor Bittner, Gerard Wiśniewski, Edyta Sienkiewicz-Szłapka, Elżbieta Łopieńska-Biernat

**Affiliations:** 1 Department of Biochemistry, Faculty of Biology and Biotechnology, University of Warmia and Mazury in Olsztyn, Oczapowskiego 1a, 10-719 Olsztyn, Poland; 2 Department of Fisheries Resources, National Marine Fisheries Research Institute, Kołłątaja 1, 81-332 Gdynia, Poland; 3 Pomorska Organizacja Producentów ARKA Sp. z o. o. [Polish Limited Liability Company], Przemysłowa 8, 81-028 Gdynia, Poland

**Keywords:** Anisakidae, drug receptors, ivermectin, oxidative stress, parasitic nematode, pyrantel

## Abstract

In the Anisakidae family, there are nematodes, most of which are parasitic for important commercial fish species. Both public health risks and socio-economic problems are attributed to these parasites. Despite these concerns, knowledge of the metabolism of these parasites remains unknown. Therefore, the main objective of this study was to investigate the receptors of drugs and oxidative metabolic status of two Anisakidae species, *Pseudoterranova decipiens* (s. s.) and *Contracaecum osculatum* (s. s.), under the influence of anthelminthic drugs, ivermectin (IVM) and pyrantel (PYR), at different concentrations: 1.56, 3.125 and 6.25 *μ*g mL^−1^ of culture medium for 3, 6, 9, 12 and 72 h. The mRNA expressions of the *γ*-aminobutyric acid receptor, acetylcholine receptor subunits, adenosine triphosphate-binding cassette transporters and antioxidative enzymes were determined. The total antioxidant capacity and glutathione *S*-transferase activity were also examined. To the best of the authors' knowledge, this is the first time that IVM and PYR have been tested against these parasitic nematodes.

## Graphical abstract

**Figure:**
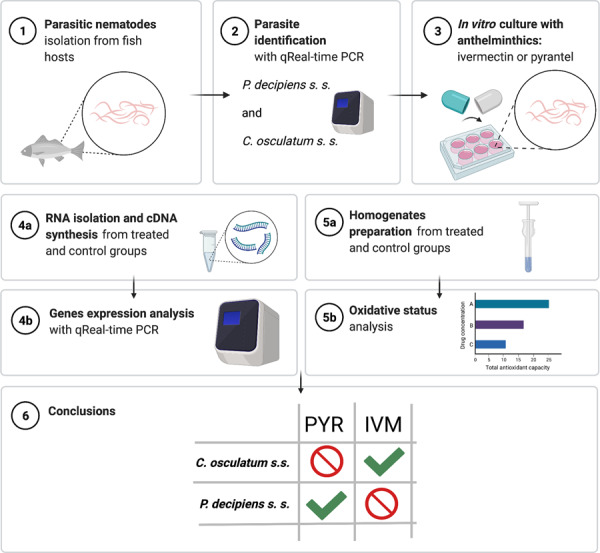

## Introduction

The parasitic nematodes of the Anisakidae family, *Anisakis simplex* (s. s.) (herring or whale worm) and *Pseudoterranova decipiens* (s. s.) (cod or seal worm), are considered the two main species that may cause anisakidosis (van Thiel, 1962; Paggi *et al*., 2000; Audicana *et al*., 2003; Baird *et al*., 2014; Buchmann and Mehrdana, 2016). Although *Contracaecum osculatum* (s. s.) (cod, seal or bird worm) may be associated with this disease, few reports recording human infections have been published (Nascetti *et al*., 1993). Anisakidosis is one of the parasitic diseases of animal origin (zoonosis) of the human digestive tract, as well as the surrounding organs in the abdominal cavity (Mattiucci *et al*., 2017; Stryiński *et al*., 2020). Recent research has revealed that the total number of worldwide anisakidosis cases caused by nematodes of the Anisakidae family was over 76 000 (up to December 2017) (Bao *et al*., 2019). *Pseudoterranova decipiens* (s. s.) and *C. osculatum* (s. s.) are pathogens usually infecting the Atlantic and Baltic cod (*Gadus morhua*), respectively (Nascetti *et al*., 1993; Paggi *et al*., 2000; Mattiucci *et al*., 2017, 2018; Levsen *et al*., 2018).

The pervasiveness of *C. osculatum* and *P. decipiens* in the Baltic Sea has increased in recent decades. The prevalence of *P. decipiens* was recorded up to 55% and intensities up to 56 worms per fish are associated with a negative correlation between worm intensity and their condition factor (Mehrdana *et al*., 2014). The prevalence of *C. osculatum* reached 100% with intensities up to 320 worms per fish, but only a slight negative correlation between intensity and fish condition factor was noted. Seals act as the final host for both worm species, and their increased occurrence during recent years has been associated with the increasing grey seal (*Halichoerus grypus*) population in the Baltic Sea area (Mehrdana *et al*., 2014). Accordingly, wild seals could be tracked down and treated with delayed-release drugs during the breeding season, after the initial test conducted in a controlled and closed group such as a zoo (Manley and Embil, 1989).

One way to combat parasitic diseases is to use pharmaceuticals on the hosts infected by the parasites. There are many groups of antiparasitic drugs, such as benzimidazoles, imidazothiazoles, tetrahydropyrimidines, macrocyclic lactones, amino-acetonitrile derivatives, spiroindoles and cyclooctadepsipeptides (Abongwa *et al*., 2017). Each group has a different way of affecting the parasite (James and Davey, 2009). In this research, the two main groups of antiparasitic drugs were studied: macrocyclic lactones and imidazothiazoles. Macrocyclic lactones [e.g. ivermectin (IVM)] affect glutamate- and *γ*-aminobutyric acid (GABA)-gated ion channels in nerve and muscle cells and increase the permeability of cell membranes for chloride ions, resulting in hyperpolarization and parasite paralysis. Imidazothiazoles [e.g. pyrantel (PYR)] affect neuromuscular transmission, as agonists of acetylcholine receptors (AChRs) increase the flow in ion channels, causing depolarization of muscle cells, resulting in spastic paralysis of the parasite. Despite the specific mechanisms of action of each group of drugs, in many parasitic nematodes, the effectiveness of anthelmintics is equivocal and varies between species. Among the various groups of parasites, the best developed xenobiotic metabolism system was found in nematodes. The biotransformation of xenobiotics, as well as endogenous compounds, is subdivided into phase I enzymes, phase II conjugation enzymes and phase III transporters. All of them serve for the detoxification of potentially harmful xenobiotics, and their activities are crucial for the effect and/or efficacy of drugs and other xenobiotics (Geerts and Gryseels, 2000). Helminths come into repeated contact with anthelmintics, and they defend themself against chemical stress by the induction of biotransformation enzymes or specific drug transporters. This induction can represent an advantageous defence strategy of the parasites and may facilitate drug-resistance development, which has become problematic in medicine and veterinary sciences (Cvilink *et al*., 2009). The development of drug resistance can be facilitated by the action of xenobiotic-metabolizing enzymes (XMEs). While human and mammalian XMEs have been intensively studied for many years, XMEs of helminth parasites have so far undergone relatively little study. The XMEs of helminth parasites may protect these organisms from the toxic effects of anthelmintics. The active transport of substrates, metabolites or conjugates through membranes mediated by special protein transporters is now considered to be the third phase of xenobiotic metabolism (Cvilink *et al*., 2009). Adenosine triphosphate (ATP)-binding cassette (ABC) transporters play a key role in the biotransformation of xenobiotics by the efflux of xenobiotic and exobiotic compounds (Leprohon *et al*., 2011). P-glycoprotein (PGP) and multidrug resistance associated proteins (MRPs) are ATP-dependent transporters involved in efflux of toxins and xenobiotics from cells. These transporters can mediate multidrug resistance (MDR) in mammalian cells, and changes in their expression and sequence are associated with drug resistance in helminths. Taken together, drug-efflux transporters and XMEs in parasitic nematodes deserve more attention and a better understanding since they seem to be crucial in helminth drug-resistance development. Moreover, knowledge of a large proportion of the metabolic pathways of anthelminthic drugs in helminths remains incomplete.

Furthermore, there are also major inter-species differences in anthelmintic metabolism between different nematode parasites. Most of the studies of anthelmintic efficacy on the nematodes of Anisakidae family were performed on the *Anisakis* spp., while the effects of the drug on the metabolism of the two remaining nematodes (*Pseudoterranova* spp. and *Contracaecum* spp.) of this family are scarce. Accordingly, it is worth emphasizing that, to the best of the authors' knowledge, this is the first report on this topic. This information could be useful for the design of new anthelmintics and, above all, in identifying other ways of nematode control. The lack of targeted anthelminthic vaccines, difficult control of the paratenic hosts (fish) and, above all, the lack of drug impact studies, underline the importance of *in vitro* screening. Moreover, many studies have shown the relationship between the expression of metabolic enzymes and drug activity and resistance (Matoušková *et al*., 2016). Transcriptomic studies will allow an assessment of the effect of an external stressor (drug) on the gene-expression profile as well as the biochemical changes (Wolstenholme *et al*., 2004; Rana and Misra-Bhattacharya, 2013) caused by this stressor. Moreover, an analysis of mRNA expression in the nematodes of genus *Anisakis* under exposure to the antiparasitic drug can provide useful information on genes associated with parasite metabolism. Gene-expression profiles could carry direct information on their role in facilitating parasite survival treated with anthelmintics as well as parasite adaptations to metabolize the chemicals (Cavallero *et al*., 2018, 2020; Łopieńska-Biernat *et al*., 2019; Palomba *et al*., 2019, 2020). Moreover, both public health risks and socioeconomic problems (Aibinu *et al*., 2019; Shamsi *et al*., 2019) are attributed to species of the Anisakidae family. Therefore, the main objective of this study was to investigate the drugs of receptors and oxidative metabolic status of *P. decipiens* (s. s.) and *C. osculatum* (s. s.) under the influence of anthelminthic drugs, IVM and PYR.

## Materials and methods

### Parasite larvae isolation and taxonomical identification

The experiment was performed on the third-stage larvae of *C. osculatum* (s. s.) isolated from Baltic cod (*G. morhua callarias*) caught in the vicinity of Władysławowo (Poland); fishery Władysławowskie, ICES square 383, subdivision 26, and the third-stage larvae of *P. decipiens* (s. s.) isolated from Atlantic cod (*G. morhua*) from a catch in the Northeast Atlantic Ocean, the fishing zone 27 2.a.2.

All larvae, i.e. 325 isolated from Baltic cod and 293 isolated from Atlantic cod, were assessed under a stereo microscope as belonging to the *Contracaecum* spp. and *Pseudoterranova* spp. species, respectively, according to methods described previously by Berland (1961, 1981) and Fagerholm (1982). No infections of fish with other parasitic species were noted. Five of the larvae isolated from each fish species were additionally subjected to molecular taxonomic identification based on the amplification of the internal transcribed spacer 1 (ITS1)/ITS2 region of genomic DNA, which was isolated with the use of Xpure™ Cell & Tissue micro (A & A Biotechnology, Gdynia, Poland) according to the manufacturer's instructions. The identification was performed using an Anis Sensitive Sniper real-time PCR kit (A & A Biotechnology, Gdynia, Poland) as described before in detail (Łopieńska-Biernat *et al*., 2020; Polak *et al*., 2020). In brief, the kit is intended to identify representatives of nematodes from the Anisakidae family found in the Baltic and North Atlantic [*A. simplex* s. s., *A*nisakis *pegreffii*, *P. decipiens* s. s., *Pseudoterranova krabbei*, *C. osculatum* s. s. and *Hysterothylacium aduncum* (a species of the Raphidascarididae family)]. The kit contains ready-to-use polymerase chain reaction (PCR) mixtures containing specific primers to identify each of the above-mentioned parasitic species, as well as a PCR mixture containing primers specific for the entire Anisakidae family (control). Seven PCRs were performed in parallel (each of the specific mixtures) with each isolated DNA sample to determine the species of the parasite by real-time PCR. A spreadsheet provided by the manufacturer after completing the *Ct* values obtained during real-time PCR shows which species' DNA was in the tested sample (File S1.1).

The rest of the larvae was prepared for the *in vitro* culture as described previously by Iglesias *et al*. (2001). In brief, larvae were rinsed thrice in a sterile saline solution (0.9% NaCl) and washed for 30 min in a bactericidal and fungicidal solution [80 mg gentamicin sulphate (010807, PPH Galfarm, Kraków, Poland), 0.625 mg amphotericin B (A9528, Sigma Aldrich, Poznań, Poland), 100 mg chloramphenicol (107464, Pharma Cosmetic, Kraków, Poland), 10 000 IU penicillin G (P3032, Sigma Aldrich, Poznań, Poland) and 4.5 mL Hanks' solution (H6648, Sigma Aldrich, Poznań, Poland) to a final volume of 10 mL saline solution].

### *In vitro* culture

All *in vitro* cultures were performed using RPMI-1640 medium (R8758, Sigma Aldrich, Poznań, Poland) enriched with 20% fetal bovine serum (F7524, Sigma Aldrich, Poznan, Poland) and 1% pepsin (P7125, Sigma Aldrich, Poznan, Poland), in a six-well plate (BD Biosciences, Warsaw, Poland), at pH = 4 (maintained with the use of 1 m HCl), 5% CO_2_ and 37°C according to the method described by Iglesias *et al*. (2001). Larvae of *C. osculatum* (s. s.) (320 individuals) and *P. decipiens* (s. s.) (288 individuals) were cultured *in vitro* for 12 or 72 h, respectively, with IVM or PYR [dissolved in 0.1% dimethylsulphoxide (DMSO); 45684, Sigma Aldrich, Poznan, Poland] at three concentrations: 1.56 (lowest), 3.125 (medium) and 6.25 (highest) *μ*g mL^−1^ of culture medium (Hu *et al*., 2013; Jones *et al*., 2015; Martin *et al*., 2020) (File S1.2). The control culture did not contain any of the tested anthelmintics (the medium had an addition of 0.1% DMSO). Larvae were examined under a stereo microscope after 3, 6, 9 and 12 h for *C. osculatum* (s. s.) (all the larvae died after that time), and additionally after 24 and 72 h for *P. decipiens* (s. s.) (all the larvae died after that time), to test the efficacy of the anthelmintics. Larvae with no mobility or contractions of the ventriculus or with alteration of the cuticle were considered dead. The concentrations of PYR and IVM at which [LC_50_ (*μ*g mL^−1^)] killed 50% of the tested groups of *P. decipiens* s. s. and *C. osculatum* s. s. were calculated and listed in Table 1.

**Table 1.** Concentrations of PYR and IVM at which tested drugs exert half of its maximal inhibitory effect [LC_50_ (*μ*g mL^−1^)] on survival of *Pseudoterranova decipiens* s. s. and *Contracaecum osculatum* s. s.

Drug	Species	
	*P. decipiens* s. s.	*C. osculatum* s. s.
PYR	5.52 (0.5)^a^	4.04 (0.5)^a^
IVM	5.34 (0.7)^a^	2.14 (0.6)^a^

a LC_50_ (*μ*g mL^−1^) was calculated for PYR and IVM after 12 h of the treatment of *P. decipiens* and *C. osculatum* with certain drug; (*r*) is linear correlation of the median-effect plot, indicating the goodness of fit; 0.5 < *r* < 0.7 indicates moderate strength of relationship; *r* > 0.7 indicates strong strength of relationship.

Larvae were collected at each time point to observe variations in gene expression and oxidative status over time. All L3 larvae of each species collected at a given time point were divided into two groups (one for RNA isolation and gene-expression studies and the second for preparation of whole body homogenates and biochemical analyses; File S1.2) and stored at −80°C for further analyses.

### Total RNA isolation, cDNA synthesis and quantitative real-time PCR

The total RNA from L3 larvae was isolated with the Total RNA Mini Plus kit (036-100, A & A Biotechnology, Gdynia, Poland) according to the manufacturer's instructions. cDNA was synthesized with 2 *μ*g RNA, oligo (dT) primers and reverse transcriptase from the TransScriba kit (4000-100, A & A Biotechnology, Gdynia, Poland). The obtained cDNA was stored at −20°C for further analysis.

The mRNA expressions of the GABA receptor, AChR subunits, ABC transporters and antioxidative enzymes were determined. Real-time PCR was carried out using a QuantStudio 3 System (Applied Biosystems, Foster City, CA, USA). The primers used in the experiment were designed using Primer3 v 0.4.0 (Untergasser *et al*., 2012) based on gene sequences in the GenBank database (http://www.ncbi.nlm.nih.gov/) (Table 2) and annealing temperatures were optimized for each (Table S1). The real-time PCR mixture contained 500 ng cDNA, 5 *μ*L Sensitive RT HS-PCR Mix SYBR^®^ (2017-1000BM, A & A Biotechnology, Gdynia, Poland), 1000 nm each primer, 0.2 *μ*L LoROX reference dye (A & A Biotechnology, Gdynia, Poland) and nuclease-free water to a final volume of 10 *μ*L. The reactions were performed in three replicates. The relative expression, presented as the fold change relative to the untreated control, as well as normalized to an endogenous reference gene (*actin*; relative quantification RQ = 1), was calculated using the comparative Pfaffl method (Pfaffl, 2001). The data were expressed as means ± standard deviation (s.d.).

**Table 2.** Primers used for quantitative real-time PCR

Gene name and NCBI accession^a^	Forward	Reverse
*unc 63* MH425578	TGCATTATCGCACACCCACT	TCGGCATTCGTGCATCAGAT
*unc 38* KM496570	GAATGTGTTCGCTCGATGGC	TACCGTTCCGAGGAAACACG
*unc 29* KM496571	GGCCAAAACAATTGCTCCGT	CGCTATCCGAGTGCGTTAGT
*arc 8* MH425576	ACAAGTGATGTCCTCGTGCA	CTGGTTCGGACATGAGAGCA
*ric 3* MG805340	ACGACCGATACGAACGCTAC	CACTGTACCTGTCGTTGCCT
*GABA 1* KP640594	GCGAGACGAGTGCTATGTCA	GGGCTTTTGGTCTCGACTCA.
*pgp-1* MG210742	TGCTAATGCTGCCGACTTCA	CTTCTCAAGCGCCTCCTGAA
*cat* MG210733	AGCCGACGGTCACAAAGATT	CACCAAGTCGATCCACGTCA
*gst* VDK26131.1	AATTCCACAAGGGCAAAGTG	CTTCCCTTCGGCATTAATCA
*sod* MG210782	AACAAAACGCATGCTGGTCC	ACATGCACAACCAACGAACG
*act* KP200883	TGGAGTGGTGCTTGACTCAG	TCACGAACAATCTCACGCTC
*ef-1α* KP326558	TCCTCAAGCGTTGTTATCTG	AGTTTTGCCACTAGCGGTT

a Full gene name: *unc 63*, acetylcholine receptor alpha-type subunit 63; *unc 38*, alpha nicotinic acetylcholine receptor subunit 38; *unc 29*, acetylcholine receptor beta subunit 29; *arc 8*, nicotinic acetylcholine receptor alpha subunit 8; *ric 3*, resistance to inhibitors of cholinesterase protein 3 gene; *GABA 1*, neurotransmitter gamma-aminobutyric acid gene; *pgp-1*, multidrug resistance protein gene; *gst*, glutathione *S*-transferase C-terminal domain-containing protein gene; *cat*, catalase gene; *sod*, superoxide dismutase gene; *act*, actin; *ef-1α*, elongation factor 1 alpha gene.

### Biochemical analyses

#### Preparation of larvae homogenates

The extract of the larvae after *in vitro* culture (10 larvae × 3 replicates of each sample) for biochemical analyses was prepared by mechanical homogenization (Omni tissue Homogenizer, Kennesaw, GA, USA) in sterile phosphate-buffered saline (pH = 7.4). The extracts were centrifuged at 4°C for 15 min (5000 ***g***) and the supernatants were then transferred into new tubes in the amount of 300 *μ*L. The protein concentration was determined using the bicinchoninic acid method (Pierce BCA Protein Assay Kit, Thermo Fisher Scientific, Waltham, MA, USA) according to the manufacturer's protocol.

#### Total antioxidant capacity

The total antioxidant capacity (TAC) was analysed by using the improved 2,2′-azinobis-(3-ethylbenzothiazoline-6-sulphonic acid) (ABTS) radical cation decolorization assay according to Re *et al*. (1999). The pre-formed radical monocation of ABTS (ABTS*+) was generated by oxidation of ABTS with potassium persulphate and was reduced in the presence of such hydrogen-donating antioxidants. Three technical replicates out of each biological replicate (drug dose *vs*  *x*  time of the culture) were performed. The results were calculated as Trolox (a water-soluble analogue of vitamin E) equivalents L^−1^.

#### Glutathione *S*-transferase activity

The glutathione *S*-transferase (GST) activity was determined using the Rice-Evans method (Rice-Evans and Miller, 1994). Enzyme activity was calculated based on the millimolar absorption coefficient (9.6 mmol^−1^ cm^−1^) for glutathione conjugate formed from 1-chloro-2,4-dinitrobenzene. Three technical replicates out of each biological replicate (drug dose *vs x* time of the culture) were performed. The GST activity was converted into arbitrary units (a.u.) per 1 mg of protein.

### Statistical analysis

The survival analysis was done using the Kaplan–Meier method (Kaplan and Meier, 1958). The survival curves were subjected to the log-rank (Mentel–Cox) test (*P* value ⩽0.05) and log-rank test for trend (*P* value ⩽0.05). The dose required to reduce nematode motility by 50% (LC_50_) was calculated for each drug using the method proposed by Tallarida (2001). Differences were considered statistically significant at *P* < 0.05. The quantitative real-time PCR data were expressed as means ± s.d. A two-way analysis of variance (ANOVA) was performed for the obtained data in GraphPad Prism 8 software (GraphPad Software Inc., San Diego, CA, USA). Differences between means were assessed by Dunnett's multiple comparisons test for expression data and Tukey's test for data obtained after biochemical analyses. Significance was defined as *P* value ⩽0.05 (*).

## Results

### Molecular identification of the species

The taxonomic identification, apart from a microscopical examination, was based on highly specific amplification of a genomic DNA fragment of the ITS1/ITS2 region. The analysis showed that all the tested larvae were belonging to the species *P. decipiens* (s. s.) and *C. osculatum* (s. s.) (File S1).

### Survival of the larvae

Larvae of *C. osculatum* (s. s.) and *P. decipiens* (s. s.) were cultured *in vitro* with IVM or PYR at three concentrations: 1.56, 3.125 and 6.25 *μ*g mL^−1^ of culture medium (lowest, middle and highest, respectively). These abbreviations will be used throughout the whole text.

#### *Contracaecum osculatum* (s. s.)

**Fig. 1.**
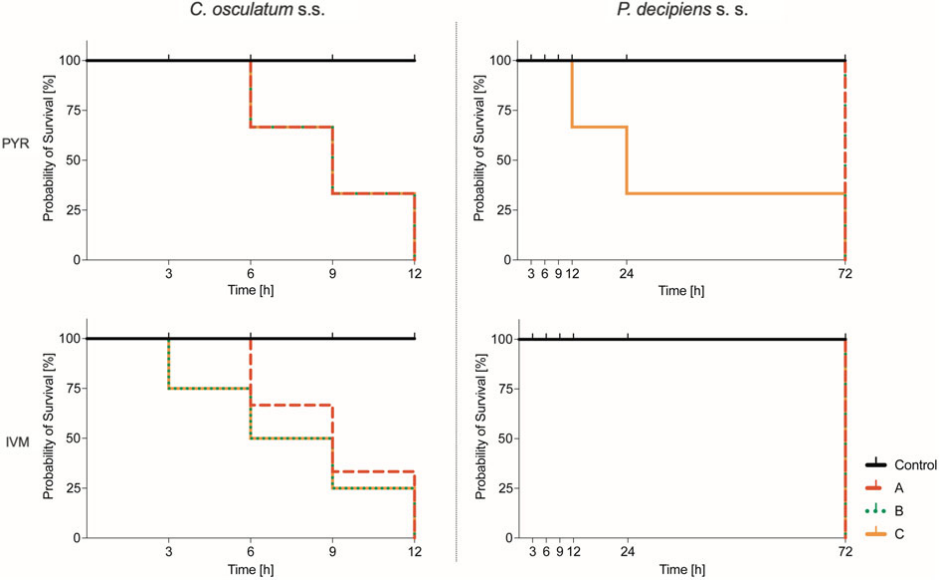
Probability of survival (%) of *Contracaecum osculatum* (s. s.) (left) and *Pseudoterranova decipiens* (s. s.) (right) larvae exposed to different concentrations (A: 1.56, B: 3.125 and C: 6.25 *μ*g mL^−1^) of PYR (top part) and IVM (bottom part) in an *in vitro* culture. A log-rank test for the trend and a log-rank (Mentel–Cox) test were performed to assess the statistically significant differences between the dose used and time (*P* value <0.05). To see detailed results, see File S2.

The survival curves were plotted to visualize the probability of survival (%) of *C. osculatum* (s. s.) L3 larvae after exposure to PYR and IVM (Fig. 1). The log-rank test for the trend showed significant results only for treatment with IVM (*P* value = 0.033; *χ*^2^ = 4.528; df = 1), which indicates that IVM decreased the mobility and survival of *C. osculatum* (s. s.) larvae cultured *in vitro* in all tested concentrations of IVM when compared to the control. However, the log-rank (Mentel–Cox) test showed (*P* value = 0.1238; *χ*^2^ = 5.761; df = 3) that the survival curves did not significantly differ from each other. This means that all the concentrations of IVM have a similar effect on the *C. osculatum* (s. s.) L3 larvae during the *in vitro* culture. The exposure to PYR did not significantly affect the survival of *C. osculatum* (s. s.) larvae (File S2). In the present study, the LC_50_ of PYR and IVM was determined to be 4.04 and 2.14 *μ*g mL^−1^, respectively, for 12 h exposure of the L3 larva of *C.* osculatum (s. s.) (Table 1).

#### *Pseudoterranova decipiens* (s. s.)

The survival curves were plotted to visualize the probability of survival (%) of *P. decipiens* (s. s.) L3 larvae after exposure to PYR and IVM (Fig. 1). The log-rank test for the trend showed significant results only for treatment with PYR (*P* value = 0.0201; *χ*^2^ = 5.4; df = 1), which indicates that PYR decreased the mobility and survival of *P. decipiens* (s. s.) larvae cultured *in vitro* in all tested concentrations of PYR when compared to the control. However, a log-rank (Mentel–Cox) test showed (*P* value = 0.09; *χ*^2^ = 6.333; df = 3) that the survival curves did not significantly differ from each other, which means that all the concentrations of PYR had a similar effect on the *P. decipiens* (s. s.) L3 larvae during the *in vitro* culture. The exposure to IVM did not significantly affect the survival of *P. decipiens* (s. s.) larvae (File S2). In the present study, the LC_50_ of PYR and IVM for a 12 h exposure of L3 larvae of *P. decipiens* (s. s.) was determined to be 5.52 and 5.34 *μ*g mL^−1^, respectively (Table 1).

### IVM treatment: actin and GABA receptor expression

**Fig. 2.**
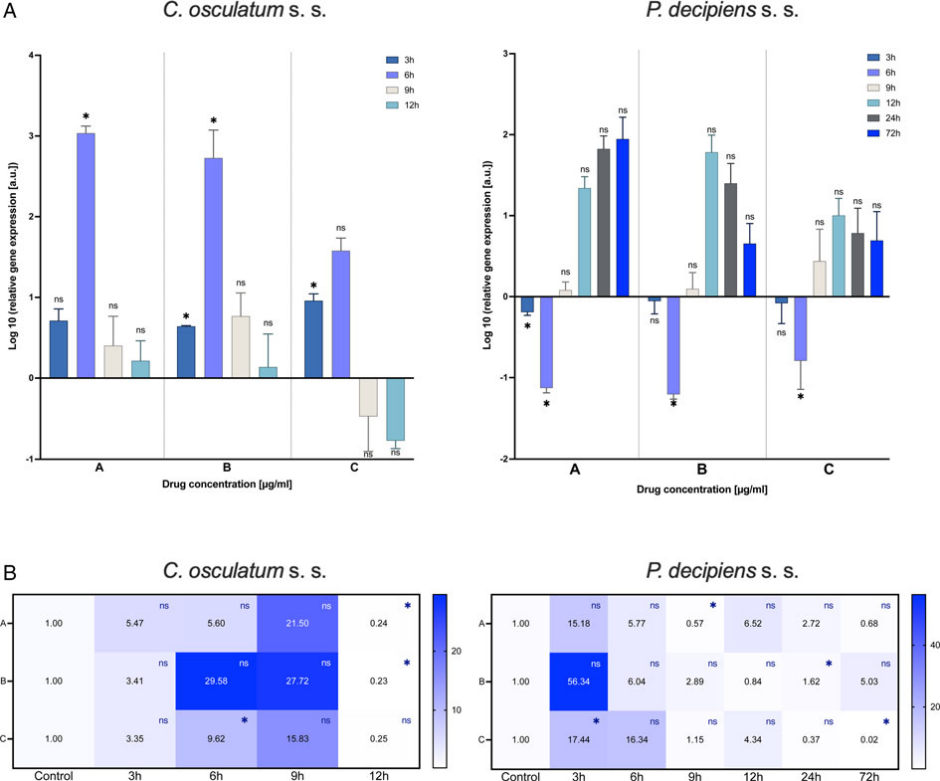
mRNA expression of *acin* gene (A) and *GABA 1* receptor (B) in *C. osculatum* (s. s.) (leftpart) and *P. decipiens* (s. s.) (right part) larvae exposed to different concentrations (A: 1.56, B: 3.125 and C: 6.25 *μ*g mL^−1^) of IVM in an *in vitro* culture. Depicted values indicate the means of three replicates ± s.d. Exact values of *GABA 1* gene expression were added in panel B. The data were presented as the fold change in gene expression normalized to an endogenous reference gene (*ef-1α*) and relative to the untreated control (relative quantification RQ = 1). A two-way ANOVA analysis was performed and the differences between means were assessed by Dunnett's multiple comparisons test. Significance was defined as *P* value ⩽0.05 (*). ns, non-significant results.

The mRNA expression of the *actin* gene was evaluated as an indicator of the tested drug's effect on muscle cells. The results show that the *actin* gene expression was upregulated by IVM in *C. osculatum* (s. s.) after 6 h of treatment in the lowest and middle doses of the tested drug (Fig. 2A). However, the expression of the receptor for IVM – *GABA 1* was significantly downregulated (in the lowest and middle doses of the tested drug) after 12 h of treatment.

The larvae of *P. decipiens* (s. s.) responded to the treatment with IVM after 6 h of treatment for the lowest and middle doses of the tested drug with downregulation of *actin* gene – in the opposite manner to *C. osculatum* (s. s.) (Fig. 2B). Moreover, significant changes in the expression of *GABA 1* receptor in *P. decipiens* (s. s.) compared to the control culture (without the drug) were noted at different times of the treatment and different doses of the used drug (3 h and highest dose; 9 h and lowest dose; 24 h and middle dose; 72 h and highest dose; Fig. 2B).

Statistical analysis showed that IVM concentration, as well as the time of the culture, had a significant influence on the expression of *actin* (File S3). Moreover, a similar effect was noted in the case of the *GABA 1* gene and *C. osculatum* (s. s.). However, in the culture with *P. decipiens* (s. s.), statistical analysis showed that there were no significant differences with *GABA 1* expression compared to larvae cultured with different drug concentrations (File S3).

### PYR treatment: actin and AChR subunit expression

**Fig. 3.**
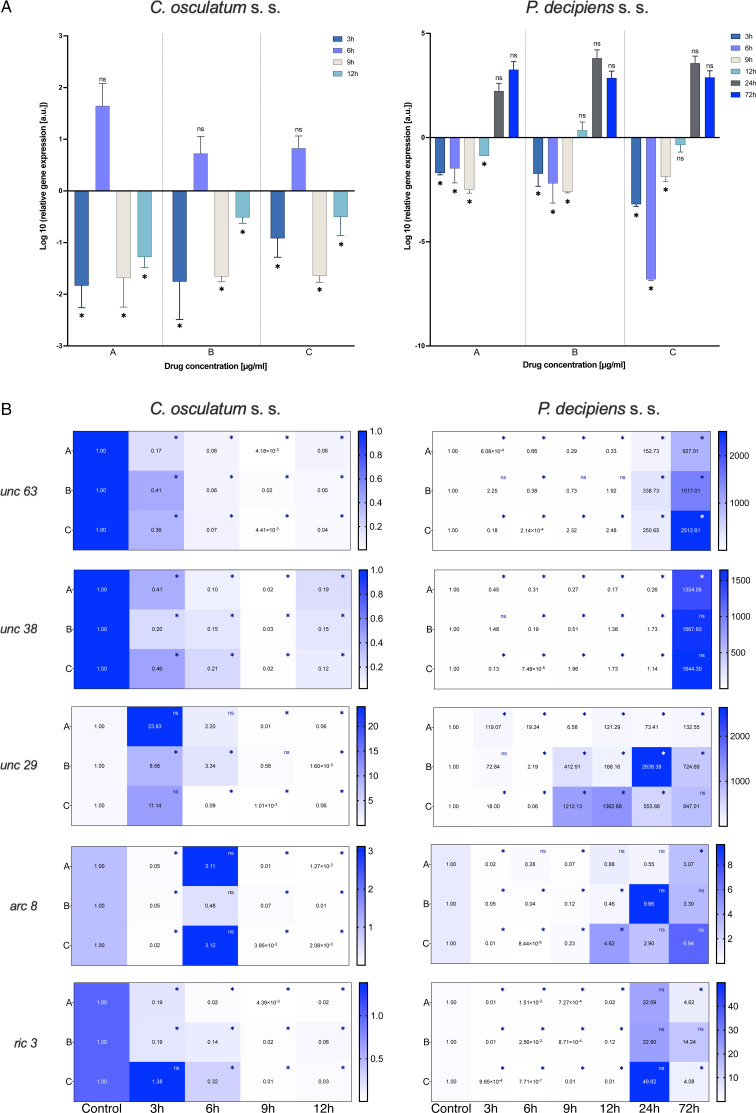
mRNA expression of *acin* gene (A) and *ACh receptor subunits* genes (B) in *C. osculatum* (s. s.) (left part) and *P. decipiens* (s. s.) (right part) larvae exposed to different concentrations (A: 1.56, B: 3.125 and C: 6.25 *μ*g mL^−1^) of PYR in an *in vitro* culture. Depicted values indicate the means of three replicates ± s.d. Exact values of *ACh receptor subunits* genes expression were added in panel B. The data were presented as the fold change in gene expression normalized to an endogenous reference gene (*ef-1α*) and relative to the untreated control (relative quantification RQ = 1). A two-way ANOVA analysis was performed and the differences between means were assessed by Dunnett's multiple comparisons test. Significance was defined as *P* value ⩽0.05 (*). ns, non-significant results.

The results of the mRNA expression of the *actin* gene showed downregulation by PYR in *C. osculatum* (s. s.) at all the used doses of the tested drug (Fig. 3A). Only after 6 h of cultivation there was no statistically significant effect on expression compared to the control sample. The larvae of *P. decipiens* (s. s.) responded to the treatment with PYR in the same manner as *C. osculatum* (s. s.) – with downregulation of the *actin* gene (Fig. 3A). However, statistically significant differences were noted after 3, 6 and 9 h of the culture with all the doses of the tested drug compared to the control (Fig. 3A). Furthermore, the statistical analysis showed that there were no significant differences between *actin* expression within the different concentrations of PYR used and within the different times of the culture for both parasitic species (File S3).

The expression of all AChR subunit genes (*unc 63*, *unc 38*, *unc 29*, *arc 8*) was downregulated after the treatment of *C. osculatum* (s. s.) with PYR compared to the control (Fig. 3B; left panel). However, the overall expression of AChRs did not differ significantly between different drug concentrations used, but significant differences were noted within the time of the culture (File S3). Similarly, the expression of the *putative chaperone resistance to inhibitors of cholinesterase 3* gene (*ric 3*) was significantly downregulated by PYR (Fig. 3B; left panel) and was also significantly different between larvae cultured with diverse drug doses and with different times of the culture (File S3).

The expression of AChRs in *P. decipiens* (s. s.) was more diverse. The *unc 63* mRNA expression was downregulated until 12 h of treatment with PYR at the lowest dose of the used drug (Fig. 3B; right panel). The expression level increased over time and after 24 and 72 h it was upregulated relative to the control. A similar situation was noted in the culture of larvae after 24 and 72 h in the middle dose of the drug. The expression of *unc 63* was upregulated after the culture with the highest dose of the PYR compared to the control.

Although the level of *unc 38* expression was mostly downregulated, upregulation was noted after 72 h in the lowest dose of the drug; 12 and 24 h in the middle dose and 9, 12 and 24 h in the highest dose of the PYR compared to the control (Fig. 3B; right panel). The expression of *unc 29* in *P. decipiens* (s. s.) was significantly upregulated after treatment with PYR. The expression of the *arc 8* subunit compared to the others was decreased. Only after 12 h of cultivation in the highest dose of the drug an increase in the expression of this subunit was noted compared to the control.

Moreover, the expression of the *ric 3* gene was significantly downregulated, while after 72 h of cultivation at extreme concentrations (lowest and highest) of the PYR, the mRNA level was higher compared to the control.

In *P. decipiens* (s. s.) different drug concentrations significantly affected the expression of *unc 63* and *unc 29*. Furthermore, comparing the expressions of AChRs and *ric 3* in different times of the culture, statistically significant differences were noted (File S3).

### Expression of ABC transporter gene

**Fig. 4.**
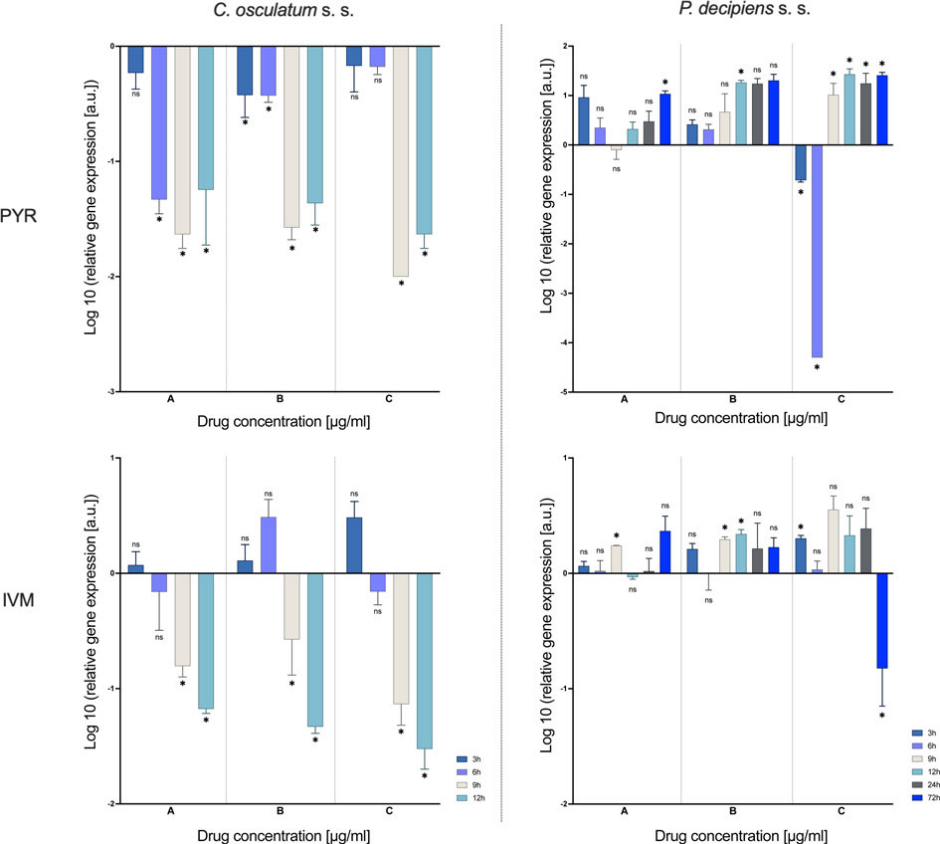
mRNA expression of ABC transporter gene *pgp-1* in *C. osculatum* (s. s.) (left part) and *P. decipiens* (s. s.) (right part) larvae exposed to different concentrations (A: 1.56, B: 3.125 and C: 6.25 *μ*g mL^−1^) of PYR (top part) and IVM (bottom part) in an *in vitro* culture. The depicted values indicate the means of three replicates ± s.d. The data were presented as the fold change in gene expression normalized to an endogenous reference gene (*ef-1α*) and relative to the untreated control (relative quantification RQ = 1). A two-way ANOVA analysis was performed and the differences between means were assessed by Dunnett's multiple comparisons test. Significance was defined as *P* value ⩽0.05 (*). ns, non-significant results.

The gene expression of an MRP *pgp-1* was evaluated as an indicator and active drug efflux from the organism of the parasite. In *C. osculatum* (s. s.) the expression of *pgp-1* was significantly downregulated after treatment with both PYR and IVM compared to the control. The mRNA level significantly decreased with time as well as with increasing drug dose in the treatment with PYR. There were no significant differences between *pgp-1* expression and IVM doses (Fig. 4, File S3).

In *P. decipiens* (s. s.), the highest dose of the PYR affected the *pgp-1* gene expression; after 3 and 6 h of the culture it was downregulated, and from 9 to 72 h it was upregulated. The IVM also affected *pgp-1* gene expression. A statistically significant upregulation compared to the control larvae treated with no drug was noted after 9 h of culture in the lowest dose of IVM, after 9 and 12 h in the middle dose and after 3 h in the highest dose. A statistically significant downregulation was noted after 72 h of culture in the highest dose of IVM compared to the control (Fig. 4). The mRNA level of *pgp-1* significantly differed between times of the culture as well as with increasing drug dose in the treatment with PYR and IVM (File S3).

### Analysis of oxidative stress markers

#### Activity of GST

The activity of GST (a phase II detoxification enzyme that catalyses the conjugation of the reduced form of glutathione to xenobiotic substrates for the purpose of detoxification) was evaluated.

**Fig. 5.**
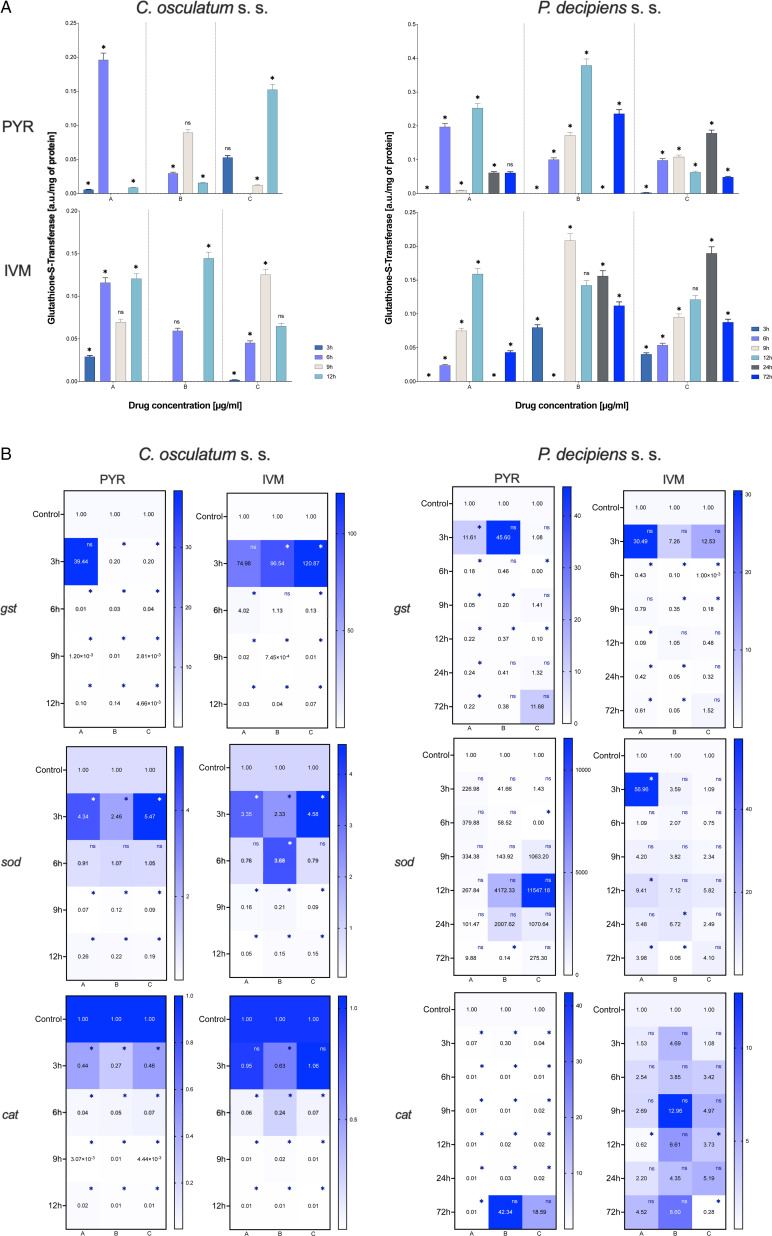
Activity of GST (A) and mRNA expression of antioxidative enzyme genes (B) in *C. osculatum* (s. s.) (left part) and *P. decipiens* (s. s.) (right part) larvae exposed to different concentrations (A: 1.56, B: 3.125 and C: 6.25 *μ*g mL^−1^) of PYR and IVM in an *in vitro* culture. The depicted values indicate the means of three replicates ± s.d. The exact values of gene expression were added in panel B. The data were presented as the fold change in gene expression normalized to an endogenous reference gene (*ef-1α*) and relative to the untreated control (relative quantification RQ = 1). A two-way ANOVA analysis was performed. Significance was defined as *P* value ⩽0.05 (*). ns, non-significant results.

In *C. osculatum* (s. s.), GST activity increased after 6 and 12 h of treatment with PYR compared to the control. Although the treatment with IVM caused similar changes in the activity of GST, significant changes were noted after 3 and 9 h in the lowest and the highest doses, respectively (Fig. 5A; left panel). In *P. decipiens* (s. s.), GST activity was increased in all the used doses of both drugs and at all culture times. The highest values were noted in the larvae treated with the middle dose of the drugs (Fig. 5A; right panel). Moreover, statistically significant differences were noted between the drug concentrations used and the times of the exposure (File S3). This confirms oxidative stress induction in the treated larvae.

#### Gene expression of antioxidative enzymes

The mRNA expression of glutathione *S*-transferase (*gst*) gene was downregulated after treatment of both parasitic species with both tested drugs (Fig. 5B). However, a statistical analysis found no significant differences in *gst* expression between *P. decipiens* (s. s.) larvae treated with different drug concentrations of PYR and IVM or between *C. osculatum* (s. s.) larvae treated with different drug concentrations of IVM (File S3).

The superoxide dismutase (*sod*) gene expression in *C. osculatum* (s. s.) was upregulated compared to the control after 3 h of PYR treatment, and 3–6 h of treatment with IVM. The *sod* expression in *C. osculatum* (s. s.) differed among the larvae cultured in different drug concentrations and among the larvae from the various times of the culture (File S3). The expression of *sod* in *P. decipiens* (s. s.) larvae treated with PYR statistically differed from the control only after 72 h in the middle dose, and after 6 h in the highest dose of the drug (Fig. 5B). Due to this, the expression did not differ between dose and time (File S3). The IVM statistically affected the groups of larvae cultured for 3, 12 and 72 h in the lowest dose and 24–72 h in the middle dose of the tested drug (Fig. 5B).

Both species of larvae responded to the PYR treatment in the downregulation of catalase (*cat*) gene expression compared to the control culture. The *cat* gene expression after IVM treatment was downregulated in *C. osculatum* (s. s.), but in *P. decipiens* (s. s.) the trend was increasing with only a few results being statistically different from the control (Fig. 5B).

#### Total antioxidant capacity

**Fig. 6.**
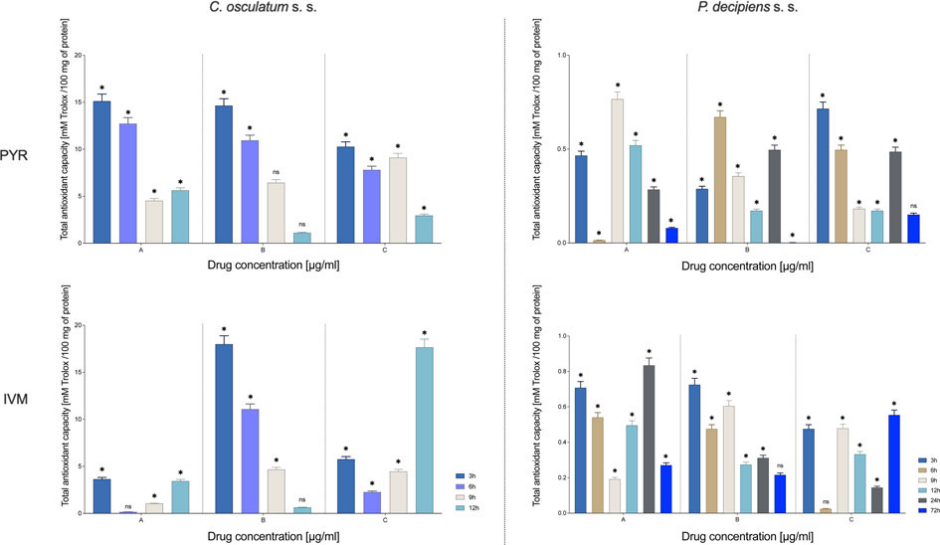
TAC of *C. osculatum* (s. s.) (left part) and *P. decipiens* (s. s.) (right part) larvae after exposure to different concentrations (A: 1.56, B: 3.125 and C: 6.25 *μ*g mL^−1^) of PYR (top part) and IVM (bottom part) in an *in vitro* culture. The depicted values indicate the means of three replicates ± s.d. The differences between means were assessed by a two-way ANOVA analysis. Significance was defined as *P* value ⩽0.05 (*). ns, non-significant results.

The TAC was significantly higher after both drug treatments (PYR and IVM) in *C. osculatum* (s. s.) and *P. decipiens* (s. s.) larvae compared to the control (Fig. 6). Moreover, statistically significant differences in TAC were noted between larvae treated with different drug concentrations and between larvae cultured for different times with both drugs (File S3). This also confirms the oxidative stress induction in the presented experimental set-up.

## Discussion

Anisakidosis is not only a problem for human infections, but also has a negative impact on the general health of fish species. Controlling infestations in the populations of marine mammals (final hosts), including grey seals in the Baltic Sea, in accordance with the complex life cycle of parasites, may be the main way to improve fish health and reduce human cases of anisakidosis (Mehrdana *et al*., 2014; Pawlak *et al*., 2019). Therefore, finding an effective and widely available pharmacotherapy is essential for the global control of anisakidosis. Understanding the mechanisms of the impact of pharmaceuticals on the parasites will allow for the selection of a targeted and effective pharmacotherapy for *in vitro* and *in vivo* tests.

Studies show that PYR and IVM act on nematode receptors and, more specifically, on ACh and GABA receptors, respectively (Feng *et al*., 2002; Williamson *et al*., 2009; Boulin *et al*., 2011; Holden-Dye *et al*., 2013; Whittaker *et al*., 2017; Martin *et al*., 2020). The main problem in the Anisakidae family is insufficient knowledge about these receptors, i.e. on their subunits, differences in the expression of genes of given receptors and on oxidative metabolism and drug transport proteins. When analysing information on other nematode species, differences in the effects of PYR and IVM on parasites can be noted. It is also worth noting that the effectiveness of drugs against parasitic nematodes is lower compared to free-living nematodes (Ros-Moreno *et al*., 1999). In the nematodes *C. osculatum* (s. s.) and *P. decipiens* (s. s.), there are no data on the configuration of the AChR and GABA building subunits and on the influence of drugs on the expression level of the genes encoding them. Therefore, the main goal of this study was to investigate the effect of two drugs PYR and IVM on the expression of genes encoding the ACh and GABA receptors and the impact on the survival of L3 larvae of the studied nematodes depending on the concentration of substances and the time of exposure.

When analysing the survival results of *P. decipiens* (s. s.), it can be concluded that PYR at the highest dose had the best effectiveness; after 24 h the probability of survival was only 30% (Fig. 1). After 12 h the value of LC_50_ for PYR was determined to be 5.52 *μ*g mL^−1^ with moderate strength of relationship of the concentration and survival (Table 1). In the case of the effects of the drug on *C. osculatum* (s. s.) larvae, no significant differences were observed between the various drug concentrations and the probability of survival, although a statistical analysis showed that IVM was more effective (Fig. 1, File S2). Also, high effect of IVM was observed with an LC_50_ value of 2.14 *μ*g mL^−1^ after 12 h (Table 1). These data are significantly different from the studies performed on L3 developmental stage of free-living *Caenorhabditis elegans*. Ferreira *et al*. showed that at concentrations from 0.01 to 1000 *μ*m after 48 and 72 h, IVM decreased the motility of *C. elegans* with increasing concentration (Ferreira *et al*., 2015). At the same time, the relationship between the activity of muscle cells and decreased expression of actin mRNA in *C. osculatum* (s. s.) should be noted (Fig. 2A). On the other hand, a different scenario of survival under the influence of drugs was observed in *P. decipiens* (s. s.) (Fig. 1).

The study of the transcriptome of the parasitic nematode *Parascaris univalens* after exposure to IVM, PYR and thiabendazole at various concentrations showed no specific response of the parasite to the drugs. Only IVM treatment at a concentration of 10^−9^ m induced expression of the gene encoding the GABA receptor subunit. However, it is worth noting that not all drugs affect the expression of genes involved in their metabolism (Martin *et al*., 2020). The studies carried out on *P. decipiens* and *C. osculatum* (s. s.) with IVM and PYR did not produce results as unequivocal as the above. Although they are nematodes from the same clade III, with more sensitive IVM receptors, after 3 and 6 h they showed similar GABA receptor expression, as noted by Feng *et al*. in *Haemonchus contortus*, a nematode from clade V (Feng *et al*., 2002). On the other hand, at 12 h, an opposite situation in the expression of GABA receptor mRNA as compared to clade V nematodes was demonstrated. Moreover, the inhibitory effects of IVM on GABA channel conductance at low concentrations of <0.2 *μ*m in *Ascaris suum*, and at higher (10 *μ*m) concentrations potentiating effects were described (Martin and Pennington, 1989). In the current study, in both *C. aduncum* and *P. decipiens*, there was no effect of IVM concentration on the expression of the GABA receptor gene (Fig. 2).

The current results indicate that at lower IVM concentrations, and after a longer exposure time, nematodes *C. osculatum* (s. s.) and *P. decipiens* (s. s.) defend against the effects of the drug (Table 1). Expression of gene encoding the GABA receptor is decreased, which may result in fewer binding sites for IVM (Fig. 2B).

Drugs from the group of imidazothiazoles (PYR) act on AChRs, causing their stimulation, resulting in spastic paralysis of the individual. Depending on the nematode species, the AChRs may differ in the activities of their subunits.

In *H. contortus*, two types of L-type AChRs have been distinguished, depending on their structure. A receptor consisting of the following subunits: Hco-UNC-29.1, Hco-UNC-63a, Hco-UNC-38 and Hco-ACR-8 is sensitive to levamisole and acetylcholine. It is the first type of L-type receptor called Hco-LAChR1. The absence of the Hco-ACR-8 subunit in the AChR classifies it as the second type of receptor type, i.e. Hco-L-AChR2, which is composed only of Hco-UNC-29.1, Hco-UNC-38 and Hco-UNC-63a. It has been shown that levamisole interacts better with the Hco-LAChR1 receptor, while PYR with Hco-L-AChR2 (Boulin *et al*., 2011). Therefore, there is a difference in the interaction of drugs with receptors between individuals of different species. Depending on the subunits that build it, the receptor may become sensitive or resistant to pharmaceuticals (Holden-Dye *et al*., 2013; Whittaker *et al*., 2017). It is worth paying attention to the research conducted by Williamson *et al*. (2009), who noted that in *A. suum* the proportions of UNC-29 (non-alpha subunit) and UNC-38 (alpha subunit) of AChR influence the parasite's response to PYR. Boulin *et al*. (2011) conducted a study on *C. elegans* in which they examined the effect of the lack of one of the AChR subunits or an ancillary protein on the activation of the ion channel. Although the lack of both the UNC-29 and UNC-38 subunits caused a rapid decrease in the receptor activity, in the absence of the UNC-38 subunit this value was lower than that in the absence of UNC-29.

The presented model of PYR action on the receptors in the two studied species from the Anisakidae family was different. In this study on *C. osculatum* (s. s.), a higher expression of *unc 29* than *unc 38* was observed in 3 and 6 h of incubation (Fig. 3B), which may indicate that in the initial period of drug treatment, there is a higher expression of L-type receptors, providing a binding site for PYR. However, after 12 h at the same concentrations, the relationship is reversed. It can therefore be assumed that the parasite defends itself against the effects of PYR in the later hours of drug exposure (Fig. 3). In contrast, *P. decipiens* (s. s.) larvae showed significant sensitivity to PYR, evident with an increasing concentration of the drug used and incubation time, by increasing the expression of *unc 29* rather than *unc 38* mRNA (Fig. 3B; File S3). A similar proportion as in *P. decipiens* (s. s.) was shown by Sarai *et al*. (2015), where they investigated changes in the expression of AChR subunits in resistant L3 of *H. contortus* larvae. At drug concentrations of 1.25 *μ*g mL^−1^, the expression of the *Hco-unc-38* subunit was twice as high, and the *Hco-unc-29* subunit was not significantly altered. In the studies performed on nematodes of the family Anisakidae, major changes were found in the expression of the gene encoding the non-alpha subunit, i.e. *unc 29* (Fig. 3B), and its expression was more stimulated than that of the alpha subunit (*unc 38*). It can be assumed that the non-alpha subunit plays a greater role in the parasite sensitivity to PYR than in free-living nematodes. Moreover, in their research, Sarai *et al*. (2015) proved that *H. contortus* individuals resistant to drugs are characterized by decreased expression of *Hco-unc-29* and *Hco-ric-3* genes. In contrast, Boulin *et al*. (2011) observed that the lack of expression of these genes prevented the creation of a functional receptor. Sarai *et al*. (2015) also focused on ancillary proteins that are required for the proper functioning of AChRs. Parasites in contact with higher drug concentrations showed significantly higher expression of ancillary proteins. Studies by the authors showed that only *P. decipiens* (s. s.) had a similar mRNA expression of the RIC3 ancillary protein (Fig. 3B).

After analysing the expression of *P. decipiens* (s. s.) genes, it was found that a higher concentration of PYR and a longer exposure time resulted in increased expression of the *ric 3* ancillary protein gene. In 24 h of incubation, together with the increased expression of the *ric 3* gene, a higher expression of the *unc 29* gene was found (Fig. 3). In this case, a parasite survival study indicated that the drug influenced nematode survival during this period. It is probable that a significant increase in the expression of both *ric 3* and *unc 29* genes makes *P. decipiens* (s. s.) larvae sensitive to PYR.

Boulin *et al*. (2011) suggested that the UNC-63 subunit in *H. contortus* is essential for the formation of the AChR. In *P. decipiens* (s. s.), when the larvae showed viability, the expression of the *unc 63* gene in the presence of PYR decreased compared to the control sample (Fig. 3). This suggests that *P. decipiens* (s. s.) expressed receptors all the time, but with a predominance of UNC-63 subunits, which allowed producing functional AChRs in 24 h PYR exposure (Fig. 3B).

In *P. decipiens* (s. s.), with the time of the exposure to the drug in the presence of the highest concentration of PYR, an increase in the expression of the *acr 8* gene (which plays pivotal role in drug sensitivity) was noted (Fig. 3). This proves that the parasites could decrease the affinity of PYR for AChR by increasing the expression of the *acr 8* gene. Observations of the expression of *P. decipiens* (s. s.) genes after cultivation in three different concentrations of PYR suggest that the increase in the concentration of the pharmaceutical in the culture medium affects the expression of genes involved in AChR activation. The changes in expression allowed reducing the affinity of PYR for the AChR.

However, in the *C. osculatum* (s. s.) at the tested PYR concentrations after 3, 9 and 12 h of the culture, a decreased amount of mRNA of the *acr 8* gene was observed (Fig. 3B). Comparing this with the *H. contortus* studies, it may be concluded that the decreased expression of the *acr 8* gene causes the formation of L2-type AChRs to be more sensitive to PYR (Boulin *et al*., 2011).

De Graef *et al*. (2013), in a study of *Cooperia oncophora*, showed the effect of IVM on the expression of phosphoglycoproteins – proteins involved in the prevention of drug absorption. Parasites exposed to IVM after 24 h showed changes in expression as a function of drug concentration. In the current research, a different scenario of the expression of these proteins was observed. In *C. osculatum* (s. s.) there was a reduction in mRNA expression during IVM and PYR treatment, which indicates the lack of receptor activation by drugs, while in *P. decipiens* (s. s.), the activation of transport proteins is very clear and indicates drug efflux depending on the dose and time of drug exposure (Fig. 4; File S3).

An interesting phenomenon is the fact that the gene expression of ABC transporters responsible for drug efflux (phase III of detoxification) is significantly reduced in *C. osculatum* (s. s.), while it increases in *P. decipiens* (s. s.), with a completely different expression profile of specific drug receptors. This may be an evidence of drug resistance (ABC transporters polymorphism) in *C. osculatum* (s. s.) or a different receptor system for both drugs (James and Davey, 2009). The results may also suggest that the *P. decipiens* (s. s.) have acquired the ability to upregulate *pgp-1* expression upon exposure to IVM and PYR.

The induction of changes in the functions of metabolic enzymes (or in pumps involved in the removal and inactivation of a drug) may trigger a mechanism of drug resistance. For example, nematodes from the Trichostrongylidae family are resistant to macrocyclic lactones in which the overexpression of genes encoding ABC transporters responsible for removing the pharmaceutical from the parasite has been observed (Raza *et al*., 2016*a*, 2016*b*, 2016*c*).

Moreover, the oxidative stress markers, i.e. TAC and GST were analysed to confirm the influence of the tested drugs on the parasitic larvae at the level of the protein. In both species of the studied nematodes, the second phase of detoxification had both high GST activity and TAC (Figs 5A and 6). The response at the transcriptional level was weak (Fig. 5B), which indicates the importance of post-translational activity and the modification of antioxidative enzymes.

Since a greater significance of the influence of drugs was noted on *P. decipiens* (s. s.) than on *C. osculatum* (s. s.), the conclusions focus mainly on *P. decipiens* (s. s.). Comparing the influence of the drugs on *P. decipiens* (s. s.), it can be concluded that the most effective drug that affects both survival and selected gene expression is PYR. The time of exposure is important and, in the case of PYR, it increases the sensitivity of the larvae to the pharmaceutical. It should be noted that lower and middle doses of PYR are less effective against the parasite but, due to that, may be less toxic to the host organism during its use. Nevertheless, the PYR in different concentrations has statistically a very similar effect on the survival of *P. decipiens* (s. s.), which is a great advantage, as hypothetically even a small dose can help fight parasites. However, when analysing selected gene expressions, the variations of the level of *P. decipiens* (s. s.) gene expression depend on the dose of the drug used. It can also be seen that *C. osculatum* (s. s.) larvae could induce drug resistance by reducing their affinity for the AChR, although further research is needed to confirm this unequivocally.

Over the next 10 years, it is expected to see a more detailed map of AChR function as well as new anthelmintics to target these receptors. In the future, the effects of drugs on other receptor subunits as well as on the abundance of ancillary proteins involved in intercellular transport and the antioxidant system should be investigated in more detail. Understanding the processes that regulate drug transfer into parasites, xenobiotic metabolisms and the sensitivity of drug's receptors is an important aspect in improving the control of parasites in human and veterinary medicine. Thus, the results presented can be used to select a suitable anthelmintic for a particular nematode species, and initial *in vivo* trials can be conducted with the final host during the breeding season in the natural environment or in zoological gardens. Moreover, knowledge of drug biotransformation mechanisms and drug resistance is necessary to select the appropriate treatment, the effective dose of the pharmaceutical and the duration of use in the fight against parasitic species.

Anisakidosis is becoming a growing problem all over the world. In addition, cases of nematode species resistant to a given drug are reported to be increasing rapidly. Due to this, it is very important to find the best solution, thanks to which a low dose of the drug, after the shortest possible time of exposure, would cause the desired result, i.e. the death of the parasite.

## Supplementary material

For supplementary material accompanying this paper visit https://doi.org/10.1017/S0031182022000543. click here to view supplementary material
