# Supplementary material for: Drug efficacy on zoonotic nematodes of the Anisakidae family: new metabolic data
Source: Parasitology. 2022 Apr 21;149(8):1065–77. doi: 10.1017/S0031182022000543 (PMC10090616; doi:10.1017/S0031182022000543)
Supplement: Supplementary file 1 [file S0031182022000543sup001.zip › S0031182022000543sup004.docx]

Supplementary Files Captions:

File S1. The results of taxonomical identification of the larvae used in the experiment (1) and detailed information about the in vitro culture and sample preparation (2).

File S2. The detailed statistics of the survival analysis of *C. osculatum* (s. s.) and *P. decipiens* (s. s.) after treatment with pyrantel and ivermectin.

File S3. The detailed statistics of the influence of drug concentration and culture time on gene/enzyme/marker in the treated larvae of *C. osculatum* (s. s.) and *P. decipiens* (s. s.).

Table S1: The temperature conditions of PCR reaction (annealing step) optimized for primers used in the study.
