# Supplementary material for: Drug efficacy on zoonotic nematodes of the Anisakidae family: new metabolic data
Source: Parasitology. 2022 Apr 21;149(8):1065–77. doi: 10.1017/S0031182022000543 (PMC10090616; doi:10.1017/S0031182022000543)
Supplement: Supplementary file 1 [file S0031182022000543sup001.zip › S0031182022000543sup005.docx]

**Table S1.** The temperature conditions of PCR reaction (annealing step) optimized for primers used in the study.

| **Gene**^1^ | ***C. osculatum* s. s.** | ***P. decipiens* s. s.** |
| --- | --- | --- |
| *unc-63* | 55-60 ^o^C | 55-60 ^o^C |
| *unc-38* | 60 ^o^C | 60 ^o^C |
| *unc-29* | 55 ^o^C | 55 ^o^C |
| *acr-8* | 55 ^o^C | 55 ^o^C |
| *ric-3* | 60 ^o^C | 60 ^o^C |
| *GABA 1* | 60 ^o^C | 60 ^o^C |
| *pgp-1* | 65 ^o^C | 65 ^o^C |
| *gst* | 60 ^o^C | 60 ^o^C |
| *cat* | 60 ^o^C | 60 ^o^C |
| *sod* | 55 ^o^C | 55 ^o^C |
| *actin* | 60 ^o^C | 60 ^o^C |
| *ef-1α* | 65 ^o^C | 65 ^o^C |

^1^ Full gene name: *unc 63* — acetylcholine receptor alpha-type subunit 63; *unc 38* — alpha nicotinic acetylcholine receptor subunit 38; *unc 29* — acetylcholine receptor beta subunit 29; *arc 8* — nicotinic acetylcholine receptor alpha subunit 8; *ric 3* — resistance to inhibitors of cholinesterase protein 3 gene; *GABA 1* — neurotransmitter gamma-aminobutyric acid gene; *pgp-1* — multidrug resistance protein gene; *gst* — glutathione-S-transferase C-terminal domain-containing protein gene; *cat* — catalase gene; *sod* — superoxide dismutase gene; *act* — actin; *ef-1α* — elongation factor 1 alpha gene.
